# Adhesive hydrogel-enabled integrative fixation for cartilage and osteochondral repair

**DOI:** 10.3389/fbioe.2026.1797388

**Published:** 2026-05-01

**Authors:** Peyman Karami, Robin Martin, Alexis Laurent, Virginie Philippe, Lee Ann Applegate, Dominique P. Pioletti

**Affiliations:** 1 Department of Orthopedic Surgery and Traumatology, Lausanne University Hospital, University of Lausanne, Lausanne, Switzerland; 2 Laboratory of Biomechanical Orthopaedics, Institute of Bioengineering, School of Engineering, École Polytechnique Fédérale de Lausanne (EPFL), Lausanne, Switzerland; 3 Manufacturing Department, LAM Biotechnologies SA, Epalinges, Switzerland; 4 Regenerative Therapy Unit, Reconstructive and Hand Surgery Service, Lausanne University Hospital, University of Lausanne, Epalinges, Switzerland; 5 Center for Applied Biotechnology and Molecular Medicine, University of Zurich, Zurich, Switzerland; 6 Oxford OSCAR Suzhou Center, Oxford University, Suzhou, China

**Keywords:** adhesive hydrogel, biomaterials sterilization, cartilage, osteochondral fixation, photo-polymerization, polymer content, tissue integration

## Abstract

Secure attachment of repair constructs to native cartilage and underlying bone tissues remains a major unmet need in chondral and osteochondral surgery. Currently existing injectable or scaffold-based cell carriers, while biologically compatible, lack the mechanical integrity and interfacial adhesion. In most cases, a secondary fixation using suturing or fibrin glue remains necessary, which can be challenging in terms of achieving long-term integration and reproducible clinical outcomes. Here, we investigated the biomechanical and adhesive performance of our previously developed photo-curable phosphoserine-functionalized methacrylated gelatin hydrogel, with the aim of defining a clinically relevant formulation window for integrative fixation. Polymer content (PC) was varied to control cohesive and interfacial properties. The hydrogel exhibited tunable biomechanical performance across formulations, with no significant batch-to-batch variability. Autoclave sterilization reduced stiffness and adhesion but preserved overall mechanical integrity, with sterilization before lyophilization (T1) causing smaller performance losses than sterilization after precursor preparation (T2). *Ex vivo* tensile adhesion on human osteochondral interfaces demonstrated multi-tissue bonding, with the hydrogel achieving several fold higher adhesion than fibrin glue on cartilage, subchondral bone, and cancellous bone. Adhesion was highest on cartilage (68.3 ± 12.5 kPa), followed by subchondral (44 ± 5.5 kPa) and cancellous bone (31.2 ± 8.2 kPa). In human chondral defects, the hydrogel could be injected and photocured under both dry and wet conditions, although surface hydration reduced adhesion by ∼50%, indicating enhanced performance under dry arthroscopy or open conditions. Cancellous bone penetration experiments revealed that lower PC (10 wt%) promotes trabecular infiltration, whereas higher PC (15 wt%) limits penetration and improves defect confinement. Overall, these results identify 15–20 wt% PC as a clinically relevant formulation window, combining injectability, manufacturing reproducibility and autoclave compatibility with robust multi-tissue adhesion. This work supports further development of the hydrogel as a potential integrative fixation strategy for minimally invasive cartilage and osteochondral repair and its future evaluation as a cell-laden delivery matrix *in vivo*.

## Introduction

Stable fixation of cartilage repair materials to the native joint surface is a persistent clinical bottleneck in cartilage and osteochondral repair (Trengove et al., 2022; Karami et al., 2024a). Biological strategies and cellular therapies such as the successive generations of autologous chondrocyte implantation (ACI), are now standard clinical practice. However, their long-term success is often compromised by mechanical failure at the interface and inconsistent integration. Membrane delamination, incomplete conformity to defect geometry and poor anchorage with the surrounding cartilage/bone are frequently reported and contribute to variable clinical outcomes (Davies and Kuiper, 2019). For surgeons, access to a reproducible and efficient delivery system to secure a cartilage construct in the defect, enhance durable healing and reduce postoperative failure rates, is a practical challenge.

Recent developments in injectable therapeutic delivery systems are encouraging to simplify cellular therapies by eliminating membranes and enabling minimally invasive delivery (Pardiwala et al., 2024). However, current clinically injectable systems are mechanically weak and poorly adhesive. Novocart® Inject, as a hyaluronan-based injectable carrier (Niemeyer et al., 2025; Niethammer et al., 2022), allows for direct defect filling, however clinical MRI follow-up has shown incomplete integration at 1 year (Niemeyer et al., 2023), in line with its weak interfacial adhesion performance (Weitkamp et al., 2023). On the other hand, delivery systems such as the Atelocollagen gel have been used clinically as a cell carrier in ACI (A-ACI) (Tohyama et al., 2009; Kato et al., 2024), but the technique consistently requires a periosteal or collagen membrane covering for fixation due to limited intrinsic adhesion and mechanical stability. Precultured scaffolds also lack the conformability and fixation stability. CaReS® delivers autologous chondrocytes embedded in a type I collagen gel that must be manually trimmed to fit the defect and glued to the underlying bone and surrounding cartilage with fibrin glue (Matsushita et al., 2022), thus the construct lacks sufficient interfacial adhesion. The failure of such carriers to provide adequate adhesion and mechanical reinforcement highlights the need for injectable materials that not only support chondrogenic activity but also establish immediate and reliable mechanical integration with surrounding tissues during early healing.

We recently developed a photo-curable adhesive hydrogel-based cell carrier designed to meet the aforementioned requirements. Our preliminary studies suggest that the hydrogel forms strong bonds with biological tissues and exhibits *in vitro* tunable physicochemical properties depending on synthesis parameters (Karami et al., 2021; Karami et al., 2024b). The hydrogel is based on a phosphoserine-functionalized methacrylated gelatin backbone. Methacrylation enables rapid photo-crosslinking and tunable mechanical properties, and phosphoserine introduces phosphate-containing groups that enhance interactions. This combination enables control over mechanical properties and interfacial interactions within a single injectable formulation. In our previous proof-of-concept work, we demonstrated its superior adhesive performance and its ability to maintain long-term lateral integration in an *in vivo* cartilage defect model (Karami et al., 2024b). These early promising findings were nevertheless obtained using animal tissue models and limited testing, without direct evaluation on human osteochondral tissues or applied assessment under manufacturing and clinically relevant constraints. Moreover, the translational potential of the material had not yet been established in terms of manufacturing reproducibility and consistency of biomechanical performance. Therefore, a more systematic evaluation of the hydrogel’s biomechanical performance and adhesion behavior on human joint tissues is essential to determine its potential as a fixation material prior to biological and *in vivo* validation. Building on our previous development of this adhesive hydrogel (Karami et al., 2021; Karami et al., 2024b), the present study aims to:

- Define the relationship between polymer content and mechanical/adhesive properties, including batch-to-batch reproducibility and sterilization effects,
- Quantify adhesion on human cartilage, subchondral bone, and cancellous bone under clinically relevant conditions, including wet arthroscopic-like environments, and
- Analyze how polymer content affects the penetration of the hydrogel in the underlying cancellous bone.

Our objective is to identify a clinically applicable formulation window for reliable fixation in minimally invasive cartilage and osteochondral repair, focusing specifically on biomechanical feasibility and interfacial adhesion on human tissues.

## Materials and methods

### Hydrogel synthesis

The hydrogel polymer was synthesized in a two-phase modification procedure with polymeric backbone methacrylation and phosphoserine functionalization according to our previous publications (Karami et al., 2021). Briefly, 10 g of the gelatin-based backbone (Sigma-Aldrich, G2500) were dissolved in 100 mL of DPBS at 60 °C under stirring for 30 min. Methacrylic anhydride (Sigma-Aldrich, 276685) was added dropwise and allowed to react for 2 h. The reaction mixture was diluted fourfold with DPBS, dialyzed against distilled water for 5 days at 50 °C and freeze-dried for 4 days to obtain the methacrylated intermediate. For phosphoserine (Flamma, 17885-08-4) modification, 0.5 g of the lyophilized polymer was dissolved in 25 mL of MES buffer (pH 5). Phosphoserine (25 mM) reaction with the modified backbone was performed using EDC/NHS (Thermo Fisher Scientific, 03450 and 24510) activation. The mixture was stirred, filtered and finally dialyzed against distilled water for 5 days. The purified polymer was then lyophilized for 4 days before use. Polymer chemical modification was validated by ^1^H NMR spectroscopy performed on a 400 MHz Bruker Avance NEO instrument. Samples consisting of 18 mg modified polymer were dissolved in 500 µL D_2_O and analyzed at 40 °C. Unmodified gelatin was characterized under the same experimental conditions. Representative spectra are included in the Supplementary Material. The lyophilized polymer was subsequently dissolved in PBS with lithium phenyl-2,4,6-trimethylbenzoylphosphinate photoinitiator (Tocris Bioscience, 6,146) to obtain precursor solutions at different polymer contents (PC) of 5, 10,15, and 20 wt%. Photo-curing was performed using visible light at a 405 nm wavelength for 30 s to obtain hydrogel samples. Three independent polymer batches were synthesized to assess the reproducibility of the process. Mechanical and adhesive properties of each batch were measured and statistical comparisons were performed to evaluate the inter-batch consistency.

### *In vitro* compression testing at different polymer contents and inter-batch reproducibility

Mechanical characterization was performed at all polymer contents using an Instron uniaxial E3000 testing system (Norwood, MA, United States). Cylindrical hydrogel samples with 6 mm diameter and 2.2 mm height were prepared in Teflon molds and subjected to unconfined compression at the rate of 0.1 mm × s^−1^ up to 25% strain (mm/mm) and the load-displacement was recorded. The compressive modulus was determined by linear interpolation of the stress-strain curve. Each formulation was tested in triplicate for each batch.

### *In vitro* adhesion testing at different polymer contents and inter-batch reproducibility

The shear adhesive strength of the hydrogel constructs was determined using a lap shear setup for all polymer contents (Rana et al., 2023). Accordingly, a 100 µL volume of the hydrogel precursor was photopolymerized between the overlapping regions of two gelatin-coated glass slides using a Teflon mold with a defined contact area of 20 × 25 mm to form a hydrogel layer adhering to both slides. The glass slides were gripped into the mechanical setup and shear adhesion measurements were conducted at a loading rate of 1 mm × s^−1^ until failure using an E3000 Instron equipped with a 50 N load cell. The adhesive strength was calculated by dividing the maximum recorded load by the bonded area. Each formulation was tested in triplicate for each batch.

### Effect of autoclave sterilization route on *in vitro* compressive and adhesive properties

For hydrogel formulations with polymer content of 5, 10 and 15 wt%, the precursor polymer in suspension was sterilized either before final lyophilization (T1) or before gelation (T2) by autoclaving at 121 °C for 10 min (see Supplementary Figure S1). After sterilizations, both compressive modulus and lap shear strength were assessed as described above, in triplicate for independent batches. In addition, the swelling ratio was measured. Cylindrical hydrogel samples were prepared by photocuring, and the initial weight of the hydrogel after synthesis (W_0_) was recorded. The samples were then immersed in PBS at 37 °C for 24 h to reach equilibrium swelling. After incubation, the samples were weighed to determine the swollen mass (W_s_). The swelling ratio was calculated as: Swelling ratio = (W_s_-W_0_)/W_0_ ×100.

### *Ex vivo* adhesion measurement on cartilage, subchondral bone plate and cancellous bone

The adhesion measurements under tensile loading were conducted on osteochondral plugs that were cut in a cylindrical shape from human allograft knee tibial plateaus (17-year-old female, 32-year-old male, 35-year-old male) in accordance with ethical regulations (n_donors = 3, n_plugs per donor = 3). The fresh-frozen allografts were obtained following meniscal transplantation (JRF Ortho, CO, United States). According to the provider’s processing protocol, all allografts were aseptically processed. After debridement and graft shaping, the tissues undergo cleansing and disinfection in an antibiotic cocktail, followed by post-disinfection sterility cultures to confirm absence of bacterial or fungal contamination. They were stored at −80 °C until use. Cylindrical osteochondral plugs were prepared from each plateau and placed in dedicated molds where the hydrogel precursor was photopolymerized on the surface. We used a previously developed custom-made adhesion setup for implementing the test (Karami et al., 2018). After curing, the hydrogel-tissue samples were gripped and the tensile load was applied with a 0.1 mm × s^−1^ rate. The adhesion strength was obtained as the maximum recorded load divided by the hydrogel-tissue bonded area. For each plug, adhesion was measured on cartilage, subchondral bone plate and cancellous bone surfaces at different polymer contents (PC) of 10 and 20 wt%. The commercial fibrin glue (Tisseel, Baxter AG, 0748627) was also used as a reference material and applied in accordance with the manufacturer’s instructions. Fibrin glue is a clinically approved two-component sealant composed of fibrinogen and thrombin that are mixed prior to application. It is commonly used in cartilage repair procedures as an auxiliary fixation material due to its biocompatibility and ease of application. However, its mechanical strength is limited and it is generally considered insufficient.

### *Ex vivo* adhesion performance in human chondral defects under dry and wet conditions

To further analyze the implementation and adhesive performance of the hydrogel, an *ex vivo* human tibial plateau model was used to demonstrate the hydrogel’s ability to anchor in a chondral defect. Two human tibial plateau allografts (25-year-old male and 31-year-old male) were used. First, two full thickness chondral defects were created and filled either a) under dry conditions, with 15 wt% hydrogel precursor cured *in situ* b) with water first, followed by 15 wt% hydrogel precursor injection through the fluid to displace it before light activation. Moreover, adhesion strength was further measured for dry and water-filled defects on both cartilage and subchondral bone under tensile loading (n_donors = 2; n_plugs per donor = 3).

### *Ex vivo* cancellous bone penetration in human osteochondral defects

To assess the influence of polymer content on hydrogel infiltration into the underlying cancellous bone of osteochondral defects, three human tibial plateaus allografts (21-year-old male, 33-year-old male, 38-year-old female) were used. To simulate osteochondral lesions in the tissue samples, cylindrical defects with a 10 mm diameter were first drilled to reach the underlying trabecular surface and the resulting plugs were removed. Two defects were created per specimen (n_donors = 3; n_defects per donor = 2). Each plateau was sectioned through the center of the defects using a precision saw and the two halves were held using a mechanical gripper. The hydrogel precursors at 10 and 15 wt% were stained with Patent Blue V dye (E131, Guerbet AG, 2379736) to enable visualization of infiltration. The precursors were applied to freshly prepared cancellous bone surfaces and after 1 min, photocuring was performed. Following curing, the hydrogel was then cut along the midline with a surgical blade and the two tissue halves were gently separated. Macroscopic images of the cross-sections were obtained to assess penetration depth. The viscosity behavior of the hydrogel precursors were characterized using a Modular Compact Rheometer 102 (Anton Paar, Peseux) equipped with a disc diameter of 25 mm. Measurements were performed under a controlled shear rate ranging from 1 to 600 s^-1^ to evaluate viscosity as a function of shear rate.

### Statistical analysis

All quantitative data are presented as mean ± standard deviation (SD). Statistical analyses were performed using two-way analysis of variance (ANOVA) with Tukey *post hoc* tests. The level of statistical significance was set at *p* < 0.05. Triplicate measurements were performed for each experimental condition. Independent polymer batches and multiple tissue donors were included to account for material and biological variability.

## Results

### *In vitro* compression and adhesive testing at different polymer contents and inter-batch reproducibility

**FIGURE 1:**
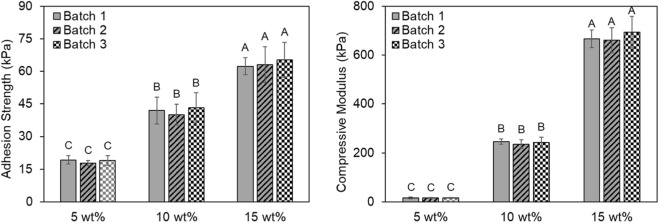
Tunable and reproducible mechanical and adhesive properties of the hydrogel. Lap shear adhesion strength (left) and compressive modulus (right) at different polymer contents of 5, 10 and 15 wt% for 3 independent polymer batch syntheses. Letters A-C represent a compact letter display of statistics wherein differences between groups labeled with the same letter are not statistically significant.

The hydrogel exhibited tunable stiffness with varying polymer contents. The results indicated a significant effect of polymer content on the hydrogel stiffness (*p* < 0.001) ranging from 16.1 ± 2.4 kPa to 673.6 ± 53.7 kPa. However, there was no significant effect of polymer batch on the compressive modulus (*p* = 0.781) confirming reproducibility of the synthesis process. Lap shear testing also showed that adhesive strength could be tuned by varying polymer content similar to the trends observed in compressive stiffness. The hydrogel exhibited a significant effect of polymer content on adhesion strength (*p* < 0.001), with mean values increasing from 18.6 ± 1.9 kPa at 5 wt% to 63.5 ± 7.2 kPa at 15 wt% polymer content. No significant effect of polymer batch was detected (*p* = 0.730). Therefore, both bulk mechanical performance and interfacial adhesion could be precisely controlled by adjusting hydrogel formulation attributes (Figure 1).

### Effect of autoclave sterilization route on *in vitro* compressive and adhesive properties at different polymer contents

We further evaluated the effect of autoclave sterilization on mechanical and adhesive properties (Supplementary Figure S2). After sterilization, both the compressive modulus and lap shear strength remained consistent across batches with no statistically significant inter-batch differences (*p* > 0.05) for all hydrogel formulations. However, sterilization itself had a significant impact on material properties (*p* < 0.001). Sterilization performed before lyophilization (T1) resulted in moderate reductions in mechanical and adhesive performance, whereas sterilization before gelation (T2) caused more pronounced losses in properties. For example, at 15 wt% polymer content, T1 sterilization reduced compressive modulus by 39.1% and lap shear strength by 33.9%, while T2 sterilization produced greater declines with decreases of 51.2% in compressive modulus and 44.6% in adhesive strength. The smaller decrease in adhesive strength compared with bulk stiffness suggests that the sterilization process affects the polymer network’s cohesion and energy dissipation capacity more strongly than its ability to form interfacial interactions with tissues. Despite these changes, the hydrogel’s mechanical integrity and adhesive capability are largely preserved through sterilization and the obtained reproducibility supports the scalability of the hydrogel manufacturing process.

### *Ex vivo* adhesion measurement on cartilage, subchondral bone plate and cancellous bone at different polymer contents

**FIGURE 2:**
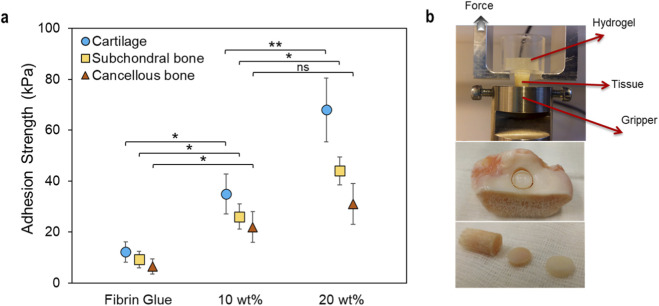
Multi-tissue adhesion mapping on human osteochondral interfaces. **(a)** Tensile adhesion strength measured on human cartilage, subchondral bone plate and cancellous bone and quantitative comparison of hydrogel formulations with fibrin glue (*n* = 3, *P < 0.05, **P < 0.01, ***P < 0.001, ****P < 0.0001). **(b)** Custom-made adhesion setup for tensile adhesion testing and sample preparation from a human tibial plateau.

To evaluate the hydrogel capacity for tissue-specific adhesion to osteochondral interfaces, we measured tensile adhesion strength on human cartilage, subchondral bone plate and cancellous bone using a custom-made mechanical setup previously developed and validated (Karami et al., 2018) (Figure 2). We selected 10 and 20 wt% polymer content, as the 5 wt% formulation, although included in the initial stiffness and shear adhesion screening, exhibited insufficient cohesive integrity and was therefore not considered clinically relevant. Conversely, the 20 wt% formulation was included to probe the upper range of adhesion performance. The hydrogel formulations were also compared to fibrin glue under similar testing conditions. Both polymer content and tissue type had significant effects on adhesion strength. The hydrogel consistently outperformed fibrin glue for all tissues and exhibited approximately 5-fold higher tensile adhesion. For instance, while fibrin glue exhibited the tensile adhesion of 12.2 ± 4.1 kPa on cartilage and 6.5 ± 3.1 kPa on cancellous bone, the 20 wt% hydrogel formulation showed substantially higher adhesion of 68.3 ± 12.5 kPa and 31.2 ± 8.2 kPa on the respective tissues. Increasing polymer content from 10 wt% to 20 wt% enhanced interfacial strength for all tissues and the highest adhesive improvement was observed on cartilage. Adhesion generally decreased from cartilage to subchondral bone plate to cancellous bone, showing the influence of substrate stiffness and surface composition on interfacial bonding.

### *Ex vivo* adhesion performance in human chondral defects under dry and wet conditions

**FIGURE 3:**
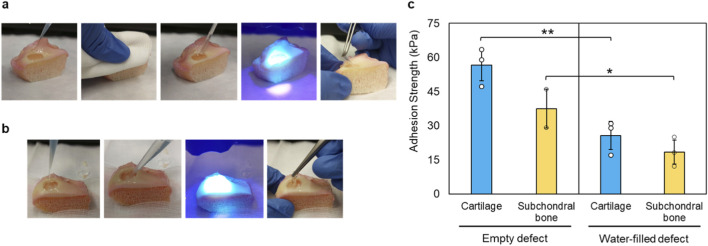
Hydrogel application and adhesion performance in an *ex vivo* human chondral defect. **(a)** Application of the hydrogel into full-thickness chondral defects (10 mm diameter) in human tibial plateau demonstrating rapid photocuring and firm fixation. **(b)** Injection through a water-filled defect simulating arthroscopic irrigation. **(c)** Quantitative adhesion strength for empty and water-filled defects on both cartilage and subchondral bone.

Cylindrical full thickness defects (10 mm in diameter) were created on the cartilage surface, filled with 15 wt% hydrogel precursor and the material was polymerized *in situ* to form an adhesive interface with the surrounding cartilage (Figure 3a). This procedure demonstrated a rapid and straightforward application of the hydrogel, as well as its fast fixation to the defect site. In a separate experiment, to approximate arthroscopic conditions where saline irrigation continuously wets the tissue surface, the defect was first filled with water and the hydrogel precursor was subsequently injected through the fluid to displace it before light activation (Figure 3b). Visual inspection after curing confirmed complete defect filling and interfacial adaptation to the surrounding tissue, but adhesion decreased compared with the application in an empty defect.

To quantitatively investigate the influence of hydration, we also measured the tensile adhesion strength measured on cartilage and subchondral bone tissues with mechanical adhesion tests (Figure 3c). When tested under wet conditions, adhesion strength decreased from 56.4 ± 6.8 kPa to 31.1 ± 5.9 kPa for cartilage and from 37.5 ± 8.5 kPa to 19.6 ± 7.9 kPa for subchondral bone. Despite this reduction, the hydrogel maintained continuous coverage of the defect and cohesive integrity. However, the approximately 50% reduction in adhesion shows the significant effect of surface hydration in interfacial bonding and indicates that the application of the hydrogel enhanced in the absence of active fluid flow during surgery, conditions that can be obtained in open surgeries and dry arthroscopic procedures. Qualitatively, only minor inter-donor variability was observed while the differences driven by tissue type and hydration state were markedly larger.

### *Ex vivo* cancellous bone penetration in human osteochondral defects at different polymer contents

**FIGURE 4:**
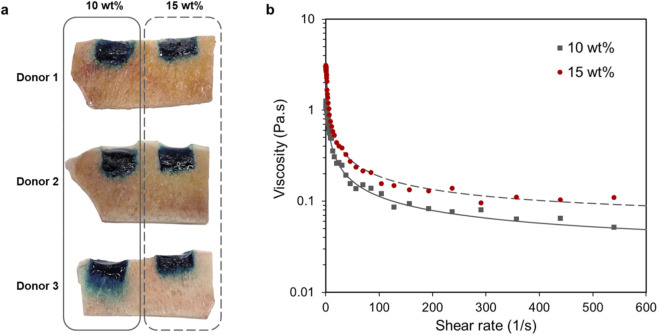
Hydrogel precursor penetration into the underlying cancellous bone. **(a)** *Ex vivo* osteochondral defects in human tibial plateau model and macroscopic images of cross-sections after *in situ* photocuring of 10 and 15 wt% hydrogels stained with a visible dye. **(b)** The viscosity behavior of the hydrogel precursors.

As an injectable hydrogel system with *in situ* photocuring, the penetration of the hydrogel precursor into the underlying bone in osteochondral defects should be clarified. Therefore, we evaluated hydrogel penetration into cancellous bone in *ex vivo* human tibial plateaus. Osteochondral defects were drilled through the subchondral plate to expose the trabecular bone and hydrogels with polymer contents of 10 wt% and 15 wt% were applied to assess the influence of viscosity on infiltration. The fluorescently labeled precursor allowed direct visualization of gel distribution within the trabecular network after photocuring. The macroscopic cross sections provided clear visualization of the hydrogel-bone interface after photo-polymerization (Figure 4).

Different behaviors were observed between formulations. The 10 wt% hydrogel precursor exhibited noticeable penetration into the trabecular structure, extending approximately 2 mm below the defect surface. In contrast, the 15 wt% formulation showed no detectable or minimal diffusion beyond the surface pores due to higher viscosity. These observations on precursor flow before polymerization suggest that a moderate viscous formulation facilitates partial infiltration with the trabecular framework, which may promote mechanical interlocking, whereas a more viscous precursor remains localized and forms adhesion with surface interactions. It should be noted that the present *ex vivo* model represents a dry air-filled cancellous environment, which likely overestimates penetration compared with physiological conditions *in vivo*, where marrow and interstitial fluid would restrict precursor diffusion. The 15 wt% and higher formulations therefore offers better defect confinement and a controlled injection procedure.

## Discussion and conclusion

Despite decades of progress in autologous chondrocyte implantation (ACI) and scaffold-based cartilage repair, rapid and stable fixation of repair constructs to native cartilage and subchondral bone remains one of the weakest steps in the current procedures. Delamination of membranes, incomplete conformity to irregular defects, and limited anchorage to the osteochondral interface continue to compromise long-term outcomes, even in third-generation ACI techniques. Existing injectable hydrogels, such as hyaluronan- (e.g., Novocart Inject) or collagen-based (e.g., CaReS, Atelocollagen gel) systems, are generally optimized for biological compatibility and chondrogenic support but lack sufficient mechanical and adhesive strength. In this context, there is a clear need for injectable adhesive systems that can establish immediate mechanical integrity and promote integrative fixation while remaining compatible with clinical workflows and manufacturing pathways.

In this study, we systematically evaluated an intrinsically adhesive, photo-curable hydrogel platform previously developed in our group (Karami et al., 2021; Karami et al., 2024b), with the specific aim of defining a formulation window compatible with the biomechanical requirements of cartilage and osteochondral fixation.

A large body of work has explored photo-crosslinkable hydrogels for cartilage repair, primarily focusing on tuning bulk mechanics and supporting chondrogenic activity (Dadsetan et al., 2007; Rice et al., 2008; Girolamo et al., 2015). Systems such as OPF-based hydrogels can achieve adjustable stiffness and sustain matrix deposition *in vitro*. Yet, they have typically not been engineered or evaluated as dedicated fixation materials at clinically relevant interfaces (Dadsetan et al., 2007). More recent generations of cartilage-oriented hydrogel systems, such as catechol-containing (Zhou et al., 2022; Yan et al., 2022), supramolecular (Li et al., 2023; Zhang et al., 2025), ECM-inspired (e.g., hyaluronic acid/gelatin-based) (Lim et al., 2020; Paul et al., 2023; Han et al., 2024; Qiu et al., 2023; Liu et al., 2017; Yang et al., 2023) or synthetic (Sharma et al., 2013; Wang et al., 2007) hydrogels have improved bonding to cartilage, but their fixation strengths or clinical practicality is limited. Beyond adhesion, physicochemical limitations, such as high swelling ratios in PEG-based systems (Karami et al., 2018; Dadsetan et al., 2007; Molina et al., 2025), may complicate integration into routine surgical procedures. In many cases, curing protocols rely on long exposure times (Karami et al., 2018; Rice et al., 2008; Paul et al., 2023), irradiation conditions (Dadsetan et al., 2007; Karami et al., 2022), or multi-component formulations (Karami et al., 2018; Paul et al., 2023; Trucco et al., 2022), representing workflow burden and translational challenges that are not compatible with intraoperative use. Furthermore, most data are generated on animal cartilage or idealized substrates, and do not account for performance loss following clinically required manufacturing and sterilization steps. Importantly, the few systems tested directly on human cartilage demonstrate even lower interfacial strength. For example, Trengove et al. reported only ∼5–6 kPa adhesion strength for a photocurable gelatin–transglutaminase hydrogel on human cartilage (Trengove et al., 2021), reflecting the additional biomechanical challenges posed by clinically relevant substrates relative to idealized bovine or porcine models. In contrast, the present hydrogel was designed from the outset as an adhesive fixation material and is tested here on human osteochondral tissues under conditions that approximate clinical use and delivery constraints.

Our results indicate that polymer content can be used to finely tune both bulk stiffness and interfacial adhesion over an order of magnitude range, without significant batch-to-batch variability. Increasing the polymer content from 5 to 15 wt% results in a substantial increase in compressive modulus and lap-shear adhesion, while preserving synthesis reproducibility. The increase in adhesion with higher polymer content can be attributed to the greater density of polymer chains and functional groups available at the interface, which increases the number of potential intermolecular interactions with tissue components. In addition, increased crosslink density enhances cohesive strength of the hydrogel, allowing more efficient stress transfer across the interface and reducing premature cohesive failure. At low polymer contents (5–10 wt%), the hydrogels exhibit limited cohesive integrity and are prone to premature failure and excessive penetration into cancellous bone when applied in osteochondral defects. Conversely, at higher contents (≥20 wt%), stiffness and adhesion are considerably enhanced, but viscosity becomes a limiting factor for injectability and controlled filling. *Ex vivo* cancellous penetration experiments highlight this balance: a 10 wt% precursor infiltrates approximately 2 mm into the trabecular network, whereas a 15 wt% formulation remains confined mainly to the defect surface. Although these experiments were performed under dry conditions that likely overestimate *in vivo* penetration in the presence of marrow and interstitial fluids, they demonstrate the importance of tuning viscosity to achieve defect confinement and predictable delivery. The intermediate formulations (15–20 wt%) therefore offer an optimal range, providing a compromise between injectability, cohesive strength, and controlled interaction with cancellous bone.

The adhesion mapping on human cartilage, subchondral bone plate, and cancellous bone confirms the multi-tissue bonding capacity of the hydrogel. The observed gradient (highest adhesion on cartilage, followed by subchondral bone plate and cancellous bone) is important. From a clinical perspective, strong adhesion to cartilage is critical to ensure lateral integration and prevent delamination at defect margins. In addition, bonding to the subchondral plate and the superficial trabecular bone provides anchorage that can stabilize constructs subjected to compressive and shear loads during early postoperative mobilization. While there is no universally accepted threshold for clinically sufficient adhesion, the several-fold improvement in adhesion relative to fibrin glue suggests that the hydrogel provides a larger safety margin against early failure at the tissue-biomaterial interface. Phosphoserine functionalization introduces phosphate groups that can contribute to interfacial interactions with extracellular matrix components. In mineralized tissues such as the subchondral bone plate, phosphate groups can exhibit affinity for calcium-containing phases. In addition, the polymeric backbone contains reactive groups that can promote interfacial contact with tissue amine groups (Karami et al., 2021). Differences in adhesion observed between cartilage, subchondral bone plate and cancellous bone are therefore likely influenced by variations in tissue composition, mineral content and surface structure, suggesting that adhesion in this system arises from a combination of physicochemical interactions and mechanical stabilization at the tissue interface.

The *ex vivo* experiments in human chondral defects further illustrate how the hydrogel behaves under application conditions relevant for surgery. Injection of the precursor into full thickness defects followed by *in situ* photocuring showed rapid defect filling, conformity to irregular surfaces and firm fixation. When defects were prefilled with water to simulate arthroscopic irrigation, the precursor still displaced the fluid and formed a continuous adhesive layer after light activation. However, adhesion strengths decreased by approximately 50% on both cartilage and subchondral bone. These results indicate that surface hydration and residual fluid at the interface significantly affect bonding, in line with known challenges of achieving robust wet adhesion. Surface hydration likely interferes with direct polymer-tissue interactions by introducing a fluid layer at the interface and diluting reactive functional groups at the contact surface. Nevertheless, even under wet conditions, adhesion remained higher than that of conventional fibrin glue-based fixation. Practically, this suggests that while the hydrogel can be applied in a wet environment, its performance is optimized under conditions where surface hydration is temporarily controlled, such as dry arthroscopy or mini-open procedures.

An important aspect of this work is the explicit consideration of manufacturing and sterilization. Autoclave sterilization is widely used in clinical settings and offers regulatory compliance, yet it can damage polymer networks and compromise material properties. By comparing two sterilization routes, before final lyophilization (T1) and after precursor preparation (T2), we show that sterilization inevitably reduces stiffness and adhesion but does not abolish the functional performance of the hydrogel. Notably, the T1 route results in more moderate decreases in compressive modulus and adhesion compared with T2, while preserving inter-batch reproducibility. The observation that stiffness is more affected than interfacial adhesion suggests that the sterilization process predominantly alters network cohesion rather than the interfacial chemistry responsible for tissue bonding. This observation suggests that autoclave exposure may partially shorten polymer chains or modify crosslink density within the bulk network, thereby reducing cohesive stiffness. However, the functional groups responsible for interfacial interactions appear to remain largely preserved, explaining why adhesive strength is comparatively less affected. These findings support selecting T1 as the preferred protocol and demonstrate that the adhesive hydrogel is compatible with standard sterilization procedures, an essential prerequisite for translation to GMP-compliant manufacturing and clinical use. Comparative evaluation with other sterilization modalities such as gamma irradiation, ethylene oxide or aseptic processing remains to be explored in future work to further assess the material performance under different clinically used sterilization conditions.

Overall, this study demonstrates the biomechanical feasibility of hydrogel systems in cartilage repair as fixation materials capable of providing immediate mechanical stabilization at the osteochondral interface. By combining tunable mechanics, multi-tissue adhesion, controlled cancellous interaction, and compatibility with autoclave sterilization, the hydrogel offers a promising strategy to advance existing cell-based therapies. Our future developments will focus on deeper characterization of the hydrogel’s long-term stability upon incorporation of human cells, as well as its performance as a cell-delivery matrix *in vivo,* to further establish its translational potential.

## Limitations and future direction

The present experiments were conducted under static loading in *ex vivo* models and should be interpreted as a biomechanical proof-of-principle for the hydrogel as a fixation material. Although it represents early postoperative conditions, it does not fully reproduce the complex mechanical and biological environment of the joint, particularly the shear forces characteristic of the patellofemoral joint. Long-term behavior under *in vivo* joint conditions and exposure to marrow components or bleeding may alter the physicochemical behavior of the implanted system. Another limitation is that the present study focused solely on the hydrogel’s biomechanical and adhesive performance without incorporating cells. Therefore, the mid- and long-term behavior of the cell-laden hydrogel should be further evaluated. While our previous large animal study demonstrated successful long-term tissue integration and functional repair (Karami et al., 2024b), further *in vivo* validation is planned to confirm the hydrogels performance as a cell carrier system for enhanced tissue healing and integration.

## Data Availability

The original contributions presented in the study are included in the article/Supplementary Material, further inquiries can be directed to the corresponding author.
