# Supplementary material for: Adhesive hydrogel-enabled integrative fixation for cartilage and osteochondral repair
Source: Front Bioeng Biotechnol. 2026 May 1;14:1797388. doi: 10.3389/fbioe.2026.1797388 (PMC13177301; doi:10.3389/fbioe.2026.1797388)
Supplement: Supplementary file 1 [file Supplementaryfile1.docx]

**Supplementary Information**

**Adhesive Hydrogel-Enabled Integrative Fixation for Cartilage and Osteochondral Repair**

Peyman Karami ^1,2 #^, Robin Martin ^1 #^, Alexis Laurent ^3,4^, Virginie Philippe ^1,4^, Lee Ann Applegate ^4,5,6^, Dominique P. Pioletti ^2*^

^1^ Department of Orthopedic Surgery and Traumatology, Lausanne University Hospital, University of Lausanne, CH-1011 Lausanne, Switzerland

^2^ Laboratory of Biomechanical Orthopaedics, Institute of Bioengineering, School of Engineering, EPFL, CH-1015 Lausanne, Switzerland

^3^ Manufacturing Department, LAM Biotechnologies SA, CH-1066 Epalinges, Switzerland

^4^ Regenerative Therapy Unit, Reconstructive and Hand Surgery Service, Lausanne University Hospital, University of Lausanne, CH-1066 Epalinges, Switzerland

^5^ Center for Applied Biotechnology and Molecular Medicine, University of Zurich, CH-8057 Zurich, Switzerland

^6^ Oxford OSCAR Suzhou Center, Oxford University, Suzhou 215123, China

^#^ Equally contribution

^*^ Corresponding author. Email: dominique.pioletti@epfl.ch


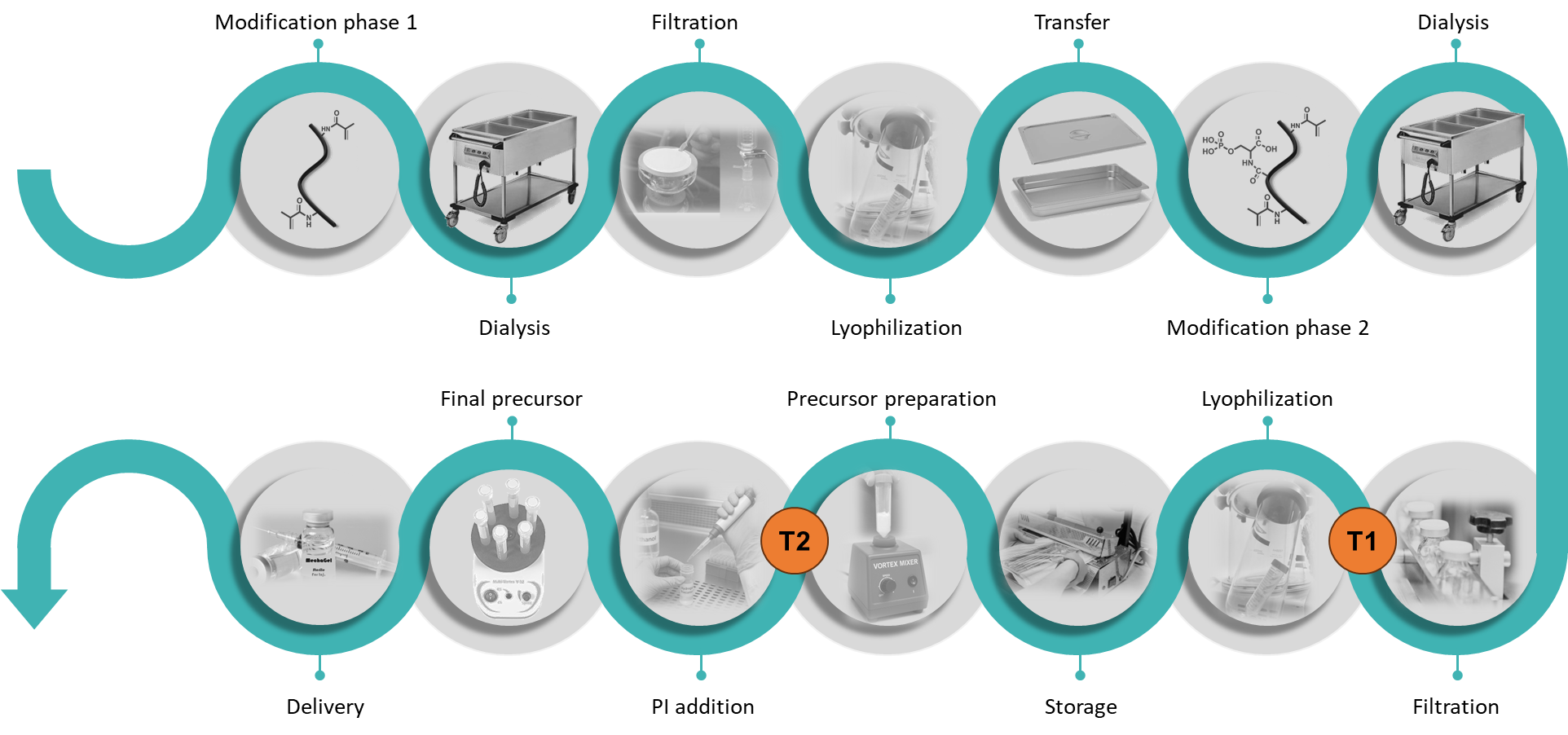


**Supplementary Figure S1.** Hydrogel manufacturing route with the two selected sterilization routs either before final lyophilization (T1) or after precursor preparation (T2).


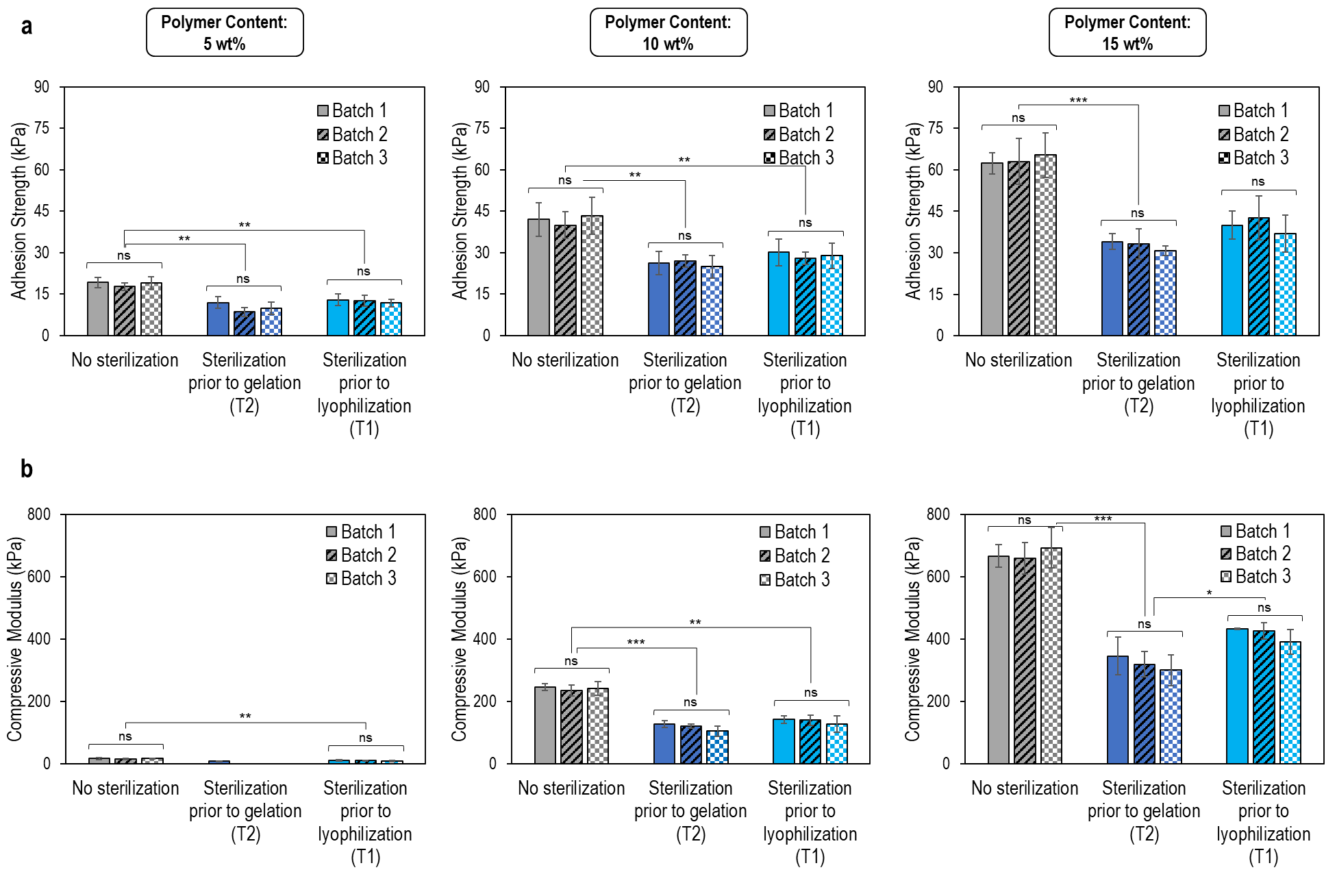


**Supplementary Figure S2.** Effect of sterilization route on hydrogel mechanical and adhesive performance and inter-batch reproducibility. (a) Lap shear adhesion strength and (b) compressive modulus at different polymer contents of 5, 10 and 15 wt% for 3 independent batches. Non-sterilized materials were compared with samples autoclaved before lyophilization (T1) and after precursor preparation (T2). (*n* = 3, **P* < 0.05, ***P* < 0.01, ****P* < 0.001, *****P* < 0.0001)

**
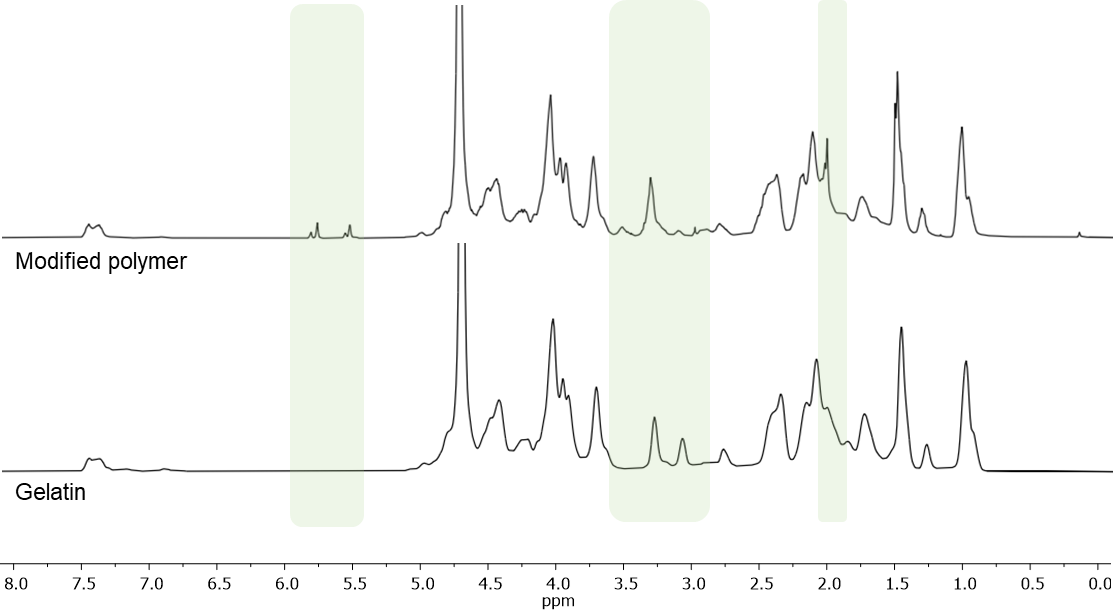
**

**Supplementary Figure S3.** Representative ¹H-NMR spectrum of modified polymeric backbone confirming methacrylation of gelatin, showing characteristic peaks corresponding to methacrylate vinyl protons.


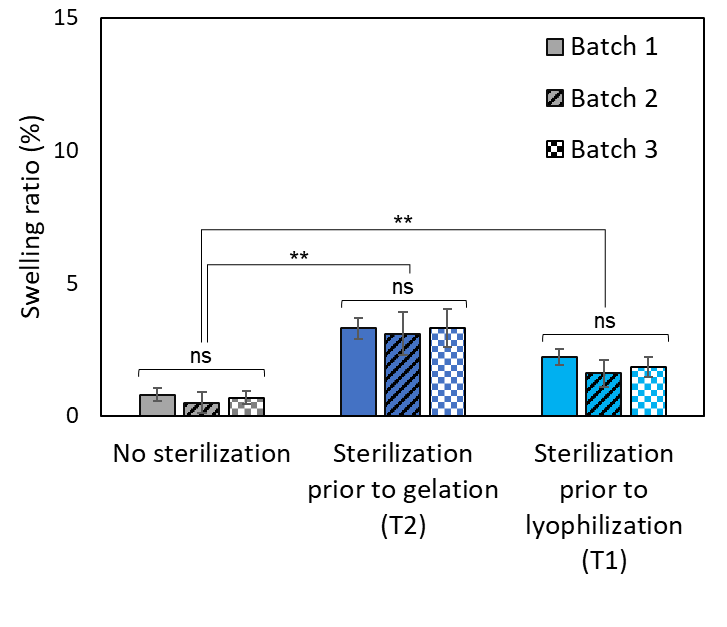


**Supplementary Figure S4.** Effect of sterilization route on swelling ratio of the hydrogel (15 wt% formulation). Non-sterilized hydrogels were compared with samples sterilized before lyophilization (T1) and before gelation (T2). Increased swelling after sterilization indicates increased water uptake after sterilization and reduced network density. However, the overall swelling remained low, indicating that the hydrogel retains a stable network structure. (*n* = 3, **P* < 0.05, ***P* < 0.01, ****P* < 0.001, *****P* < 0.0001)
